# Liquid‐based cell suspension of supraclavicular lymph node fine‐needle aspirate as an alternative specimen for NGS‐based genomic profiling in advanced lung cancer

**DOI:** 10.1002/ctm2.196

**Published:** 2020-10-06

**Authors:** Xun Zhang, Ying Chen, Li Chu, Jianjiao Ni, Analyn Lizaso, Bing Li, Xiaoqian Guan, Hao Liu, Guodong Li, Zhengfei Zhu

**Affiliations:** ^1^ Department of Ultrasound Diagnosis Fudan University Shanghai Cancer Center Shanghai China; ^2^ Department of Oncology, Shanghai Medical College Fudan University Shanghai China; ^3^ Department of Pathology Fudan University Shanghai Cancer Center Shanghai China; ^4^ Department of Radiation Oncology Fudan University Shanghai Cancer Center Shanghai China; ^5^ Burning Rock Biotech Guangzhou China; ^6^ Department of Intervention Radiology Fudan University Shanghai Cancer Center Shanghai China; ^7^ Institute of Thoracic Onology Fudan University Shanghai China

**Dear Editor,**

Our study demonstrates the clinical utility of liquid‐based cell suspension of fine‐needle aspirates (FNA) of supraclavicular lymph node (SLN) stored in 10% neutral buffered formalin (NBF) as a source of tumor‐derived DNA for next‐generation sequencing (NGS)‐based mutation profiling and guides therapeutic decisions of patients with advanced lung cancer.

Over the past decade, various targeted therapies have dramatically improved the survival of patients with advanced lung cancer harboring actionable genetic mutations, making it essential to implement NGS‐based molecular profiling in clinical practice to simultaneously interrogate and identify potential tyrosine kinase inhibitor‐sensitizing mutations for treatment planning.^1^, ^2^, ^3^ The use of specimens obtained in a less invasive manner, including cytology specimens collected through FNA biopsy has been extensively explored for NGS assays.^2^, ^4^, ^5^, ^6^ SLN metastasis occurs in 26‐42% of advanced lung cancer.^7^ SLN‐FNA prepared as cytospin or formalin‐fixed paraffin‐embedded (FFPE) cell blocks have been reported in PCR‐based EGFR mutation testing.^8^ Compared to conventional smears, liquid‐based cytology maintains the morphology of the collected cells, which preserves DNA integrity and minimizes DNA degradation, thereby improving the quantity and quality of DNA extracted for subsequent molecular assays.^9^ Moreover, liquid‐based cytology stored in 10% NBF is more convenient for molecular testing applications than FFPE cell blocks by skipping the laborious paraffinization/deparaffinization sample processing steps.

SLN‐FNA samples stored in 10% NBF were obtained from 54 lung cancer patients with pathologically confirmed metastatic SLNs diagnosed between January 2018 and December 2019 at Fudan University Shanghai Cancer Center, 53 of whom also had paired plasma samples (Figure 1A). The baseline characteristics of these patients were listed in Table S1. DNA from the SLN‐FNA samples and plasma samples were, respectively, extracted according to the manufacturer's protocol using QIAamp kits for DNA FFPE tissue and circulating nucleic acid extraction (Qiagen, Hilden, Germany). All samples passed quality control for subsequent NGS library construction assessed through high‐sensitivity DNA assay in Agilent 2100 Bioanalyzer (Agilent, CA, USA). NGS libraries were prepared from SLN‐FNA DNA and circulating cell‐free DNA from paired plasma samples using targeted panels interrogating 168 and 520 cancer‐related genes from Burning Rock Biotech and sequenced on Nextseq500 (Illumina, CA) with paired‐end reads at target sequencing depths of 1,000× for SLN‐FNA and 10,000× for plasma samples as previously described.^6^ Paired leukocyte genomic DNA was sequenced to filter out clonal hematopoiesis‐related mutations. The 520‐gene panel also interrogates the same genomic regions for the genes included in the 168‐gene panel.

**FIGURE 1:**
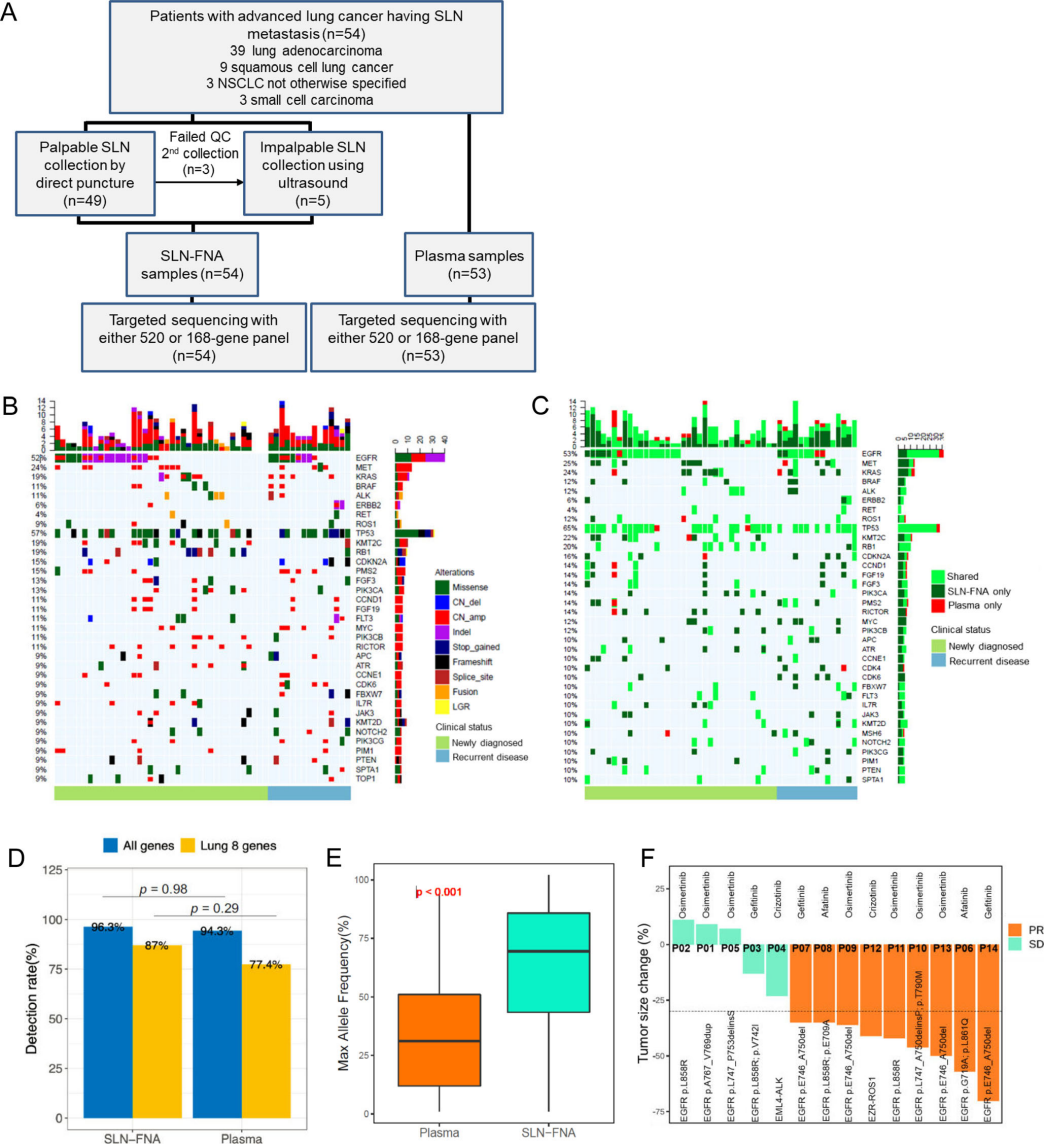
Feasibility and clinical utility of SLN‐FNA suspended in 10% NBF as a liquid‐based cytology specimen for NGS‐based genomic profiling in advanced lung cancer. (A), Flow diagram illustrating the study design. (B) and (C), Oncoprints summarizing the (B) mutations derived from SLN‐FNA of each of the 54 patients with advanced lung cancer considering only the genes from the 168‐gene panel. Different types of mutations were represented by different colors. (C) Comparison of mutations derived from matched SLN‐FNA and plasma samples in 51 patients with advanced lung cancer. The patients without plasma sample (n = 1) and no detected mutations from the gene panel (n = 2) were not included in this oncoprint. Mutations detected in both SLN‐FNA and plasma samples were shown in bright green (shared). Mutations that were only detected from the plasma (PLA) or SLN‐FNA were shown in red and dark green, respectively. Each row represents a gene. Top bar denotes the number of mutations detected in each patient. Side bar represents number of patients with mutation in a certain gene. The mutation profile was arranged according to the clinical status of the patient either initially diagnosed (light green, (B), n = 39; (C), n = 36) or recurrent disease (light blue, n = 15). (D) Bar plot illustrating the comparable mutation detection rates between SLN‐FNA and plasma samples in the 168 genes and the eight lung cancer oncogenic genes and (E) box plot illustrating the significantly higher maximum allele frequency (maxAF) of mutations detected from SLN‐FNA as compared to plasma samples of 53 patients with advanced lung cancer. (F) Waterfall plot illustrating the overall response rate of the 14 patients who received targeted therapy. Green represents stable disease. Orange represents partial response. The lower dashed line indicates the 30% tumor reduction from baseline, which is the lower limit of partial response as per RECIST criteria. However, the patient (P41) progressed soon after the first evaluation of treatment response, and regarded as progressive disease

SLN‐FNA samples attained an actual average sequencing depth of 1763×. A total of 478 mutations were detected in 124 genes from the SLN‐FNA samples from 52 patients, yielding a detection rate of 96.3% (Figure 1B). A total of 310 mutations were detected in 106 genes from the plasma samples of 50 patients, yielding an overall concordance rate of 94.3% with SLN‐FNA samples (Figure 1C). Table 1 summarizes the distribution of single nucleotide variants (SNVs), small insertion or deletion (Indels), copy number variants (CNVs), and fusions detected from the SLN‐FNA and plasma samples. The mutation detection rates for 168 genes (96.3% (52/54) vs 94.3% (50/53); *P* = .98) and eight actionable genes in lung cancer (87.0% (47/54) vs 77.4% (41/53); *P* = .29) were not statistically different between SLN‐FNA and plasma samples (Figure 1D, Table S2). SLN‐FNA was found to be significantly superior to plasma in detecting CNV for the genes included in the gene panels (63.0% (34/54) vs 20.7% (11/53); *P* < .001), and in the eight genes (46.3% (25/54) vs 11.3% (6/53); *P* < .001). Similarly, the maximum allele fraction of mutations from SLN‐FNA samples, particularly SNV and Indels, were significantly higher as compared to plasma samples (*P* < .001; Figure 1E). Tumor mutation burden (TMB) analyzed for SLN‐FNA samples of 35 patients sequenced using the 520‐gene panel revealed a median TMB of 4.8 mutations/Mb, ranging from 0.0 to 13.5 mutations/Mb.

**TABLE 1:** Comparison between the number of mutations detected from matched SLN‐FNA and plasma samples of the 54 patients with advanced lung cancer

	Total number of mutations		Number of mutations				
Mutation types	SLA‐FNA	Plasma	Shared	SLN‐FNA only	Plasma only	Concordance rate (%)	*P*‐value
168 genes							0.68
SNV+Indels	221	216	193	28	23	79.1%	
CNV	193	42	28	165	14	13.5%	
Fusions	6	6	6	0	0	100%	
Eight driver genes							0.21
SNV+Indels	47	48	43	4	5	82.7%	
CNV	38	10	8	30	2	20%	
Fusions	6	6	6	0	0	100%	

Abbreviations: CNV, copy number variation; Indels, small insertion or deletions; SLN‐FNA, supraclavicular lymph node fine needle aspirate; SNV, single nucleotide variation.

Importantly, 14 patients who received targeted therapy based on the NGS results from the SLN‐FNA samples achieved an overall response rate of 64.3% and a disease control rate of 100%, based on the Response Evaluation Criteria In Solid Tumors (RECIST) v.1.1 (Figure 1F, Table S3), which is consistent with reported efficacy for targeted therapies for EGFR, ALK, and ROS1 according to tissue‐based molecular assay results.^1^, ^2^, ^3^ Notably, a patient detected with a rare *EZR‐ROS1* fusion from his SLN‐FNA sample achieved partial response with front‐line crizotinib therapy (Figure S1). In addition to patients with non‐small‐cell lung cancer, we also investigated the mutation profile of three patients with small‐cell lung cancer (SCLC). Of them, two patients were detected with concurrent *TP53* and *RB1* mutations, which are typical molecular features of SCLC.^10^ The remaining patient harbored concurrent *TP53* p.Arg342* and *PIK3CA* p.Glu542Lys, which were detected from both his plasma and SLN‐FNA samples.

To our knowledge, this study is the first to demonstrate the utility of liquid‐based cell suspension of SLN‐FNA as an alternative specimen for NGS‐based genomic profiling in patients with advanced lung cancer. The samples obtained through SLN‐FNA stored in 10% NBF provided sufficient quantity and satisfactory quality of tumor DNA. NGS analysis of SLN‐FNA specimens allowed the reliable detection of classic as well as rare mutations of different mutation types including SNV, Indels, CNV, and gene rearrangements, which also enable reliable estimation of TMB. Comprehensive mutational profile derived from SLN‐FNA specimens could provide useful molecular information to guide therapeutic decisions in patients with lung cancer of various histology (i.e., adenocarcinoma, squamous cell, SCLC, and others), disease status (i.e., initially diagnosed or recurrent disease), and treatment status (e.g., treatment‐naïve or prior‐treated) who have SLN metastasis. Future studies comparing matched primary tumor tissue and SLN‐FNA samples with a larger patient size are warranted.

## CONFLICT OF INTEREST

Analyn Lizaso, Bing Li, Xiaoqian Guan, and Hao Liu are employed by Burning Rock Biotech. The other authors declare no potential conflict of interest.

## AUTHOR CONTRIBUTIONS

Xun Zhang: conceptualization, data curation, investigation, formal analysis, writing—review and editing; Ying Chen: conceptualization, data curation, validation, methodology, resources, formal analysis, writing—review and editing; Li Chu: data curation, investigation, validation, writing—review and editing; Jianjiao Ni: data curation, methodology, writing—review and editing; Analyn Lizaso: formal analysis, writing (original draft), writing—review and editing; Bing Li: visualization, software, writing—review and editing; Xiaoqian Guan: visualization, writing—review and editing; Hao Liu: project administration, writing—review and editing; Guodong Li: conceptualization, project administration, supervision, writing—review and editing; Zhengfei Zhu: conceptualization, funding acquisition, project administration, supervision, writing—review and editing.

## CONSENT FOR PUBLICATION

All the authors provided consent for publication.

## Supporting information


**Supplementary Table S1**. Clinicopathologic characteristics of the cohort
**Supplementary Table S2**. Mutation in the eight classic lung cancer oncogenic driver genes detected from the SLN‐FNA and plasma samples of the cohort
**Supplementary Table S3**. Clinical summary of the 14 patients who received targeted therapy

**Supplementary Figure S1**. Treatment efficacy of crizotinib in a patient with lung adenocarcinoma harboring *EZR‐ROS1* rearrangement. Computed tomography scans of Patient P12 at baseline (February 11, 2019) (A), and tumor responses at 6 months (September 20, 2019) of crizotinib therapy (B). C. Illustration of *the EZR‐ROS1* rearrangement detected from the SLN‐FNA sample of the patient. Integrated Genome Viewer screenshot showing the adjoining intron 10 of *EZR* at chromosome 6: 159,191,625 to intron 10 of *ROS1* at chromosome 6:117,646,698, which retains the ROS1 intracellular tyrosine kinase domain. Each gray row represents the sequencing read from a DNA fragment. Bottom bar shows the DNA sequence annotation of *ROS1* (left) and *EZR* (right).

## Data Availability

All authors confirm adherence to the policy. The data that support the findings of this study are available from the corresponding author upon reasonable request.
