# Supplementary material for: Liquid‐based cell suspension of supraclavicular lymph node fine‐needle aspirate as an alternative specimen for NGS‐based genomic profiling in advanced lung cancer
Source: Clin Transl Med. 2020 Oct 6;10(6):e196. doi: 10.1002/ctm2.196 (PMC7536615; doi:10.1002/ctm2.196)
Supplement: Supplementary file 1 — Supplementary Table S1. Clinicopathologic characteristics of the cohort Supplementary Table S2. Mutation in the eight classic lung cancer oncogenic driver genes detected from the SLN‐FNA and plasma samples of the cohort Supplementary Table S3. Clinical summary of the 14 patients who received targeted therapy [file CTM2-10-e196-s002.docx]

**Table S1**. Clinicopathologic characteristics of the cohort

| Clinicopathologic characteristics | n (%)  n=54 |
| --- | --- |
| Gender |  |
| Male | 31 (57.4%) |
| Female | 23 (42.6%) |
| Age (years) (median [range]) | 60.5 [35-83] |
| Clinical stage |  |
| IIIB | 11 (20.4%) |
| IIIC | 5 (9.2%) |
| IVA | 7 (13.0%) |
| IVB | 31 (57.4%) |
| Histology |  |
| Adenocarcinoma | 39 (72.2%) |
| Squamous cell carcinoma | 9 (16.7%) |
| Non-small cell lung cancer, not otherwise specified (NOS) | 3 (5.6%) |
| Small cell carcinoma | 3 (5.6%) |
| Prior therapy |  |
| None | 39 (72.2%) |
| Targeted therapy | 6 (11.1%) |
| Chemotherapy alone | 5 (9.3%) |
| Chemotherapy + radiotherapy | 4 (7.4%) |

**Table S2**. Mutation in the 8 classic lung cancer oncogenic driver genes detected from the SLN-FNA and plasma samples of the cohort

| Mutations | Number of mutations | | |
| --- | --- | --- | --- |
|  | Detected in both SLN-FNA and plasma | Detected in SLN-FNA only | Detected in Plasma only |
| *EGFR* all mutations | 36 | 5 | 3 |
| *ERBB2* amplification | 0 | 2 | 0 |
| *MET* | 3 | 9 | 1 |
| *MET* amplification | 2 | 9 | 0 |
| *MET* exon 14 skipping | 0 | 0 | 0 |
| *BRAF* V600E | 0 | 1 | 0 |
| *KRAS* G12X | 2 | 0 | 0 |
| *ROS1* fusion | 1 | 0 | 0 |
| *ALK* fusion | 3 | 0 | 0 |
| *RET* fusion | 1 | 0 | 0 |
| Total | 48 | 26 | 4 |

**Table S3**. Clinical summary of the 14 patients who received targeted therapy

| Patient ID | Gender | Age | Histology | Clinical stage | Actionable mutation detected | Allelic fraction | | Sequencing quality control status | Targeted therapy | | |
| --- | --- | --- | --- | --- | --- | --- | --- | --- | --- | --- | --- |
|  |  |  |  |  |  | SLN-FNA | Plasma |  | Specific therapy administered | Therapy line | Best response |
| P1 | Female | 62 | adenocarcinoma | IVB | *EGFR* A767_V769dup | 34.04% | 0.36% | Passed | osimertinib | 1L | SD |
| P2 | Female | 62 | adenocarcinoma | IVB | *EGFR* L858R | 18.46% | 3.66% | Low | osimertinib | 1L | SD |
| P3 | Male | 49 | squamous cell carcinoma | IVB | *EGFR* L858R, *EGFR* V742I | 33.73%, ND | 0.24%, 0.6% | Passed | gefitinib | 1L | SD |
| P4 | Male | 39 | adenocarcinoma | IVB | *EML4-ALK* | 95.4% | 37.91% | Passed | crizotinib | 1L | SD |
| P5 | Female | 51 | adenocarcinoma | IVB | *EGFR* L747_P753delinsS | 36.20% | 2.43% | Passed | osimertinib | 1L | SD |
| P6 | Male | 68 | adenocarcinoma | IIIB | *EGFR* G719A, *EGFR* L861Q | ND, 21.98% | 0.18%, 0.66% | Low | afatinib | 1L | PR |
| P7 | Female | 72 | adenocarcinoma | IVA | *EGFR* E746_A750del | 59.79% | 5.21% | Passed | gefitinib | 1L | PR |
| P8 | Female | 54 | adenocarcinoma | IVB | *EGFR* L858R, *EGFR* E709A | 59.34%, 59.07% | NA | Passed | afatinib | 1L | PR |
| P9 | Female | 54 | adenocarcinoma | IVB | *EGFR* E746_A750del | 65.58% | 3.54% | Low | osimertinib | 1L | PR |
| P10 | Male | 67 | adenocarcinoma | IVB | *EGFR* L747_A750delinsP, *EGFR* T790M | 77.02%, 50.13% | 30.96%, 14.55% | Passed | 1L gefitinib;  2L osimertinib | 2L | PR |
| P11 | Male | 71 | adenocarcinoma | IVB | *EGFR* L858R | 57.07% | 3.70% | Passed | osimertinib | 1L | PR |
| P12 | Female | 51 | adenocarcinoma | IIIC | *EZR-ROS1* | 26.98% | 0.37% | Passed | crizotinib | 1L | PR |
| P13 | Female | 46 | adenocarcinoma | IVB | *EGFR* E746_A750del | 22.67% | 6.62% | Low | osimertinib | 1L | PR |
| P14 | Male | 67 | adenocarcinoma | IVB | *EGFR* E746_A750del | 55.81% | 6.64% | Passed | gefitinib | 1L | PR |

Abbreviations: ND, not detected; 1L, first-line; 2L, second-line; PR, partial response; SD, stable disease; SLN-FNA, supraclavicular lymph node-fine needle aspirate
