# Supplementary material for: Liquid‐based cell suspension of supraclavicular lymph node fine‐needle aspirate as an alternative specimen for NGS‐based genomic profiling in advanced lung cancer
Source: Clin Transl Med. 2020 Oct 6;10(6):e196. doi: 10.1002/ctm2.196 (PMC7536615; doi:10.1002/ctm2.196)
Supplement: Supplementary file 2 — Supplementary Figure S1. Treatment efficacy of crizotinib in a patient with lung adenocarcinoma harboring EZR‐ROS1 rearrangement. Computed tomography scans of Patient P12 at baseline (February 11, 2019) (A), and tumor responses at 6 months (September 20, 2019) of crizotinib therapy (B). C. Illustration of the EZR‐ROS1 rearrangement detected from the SLN‐FNA sample of the patient. Integrated Genome Viewer screenshot showing the adjoining intron 10 of EZR at chromosome 6: 159,191,625 to intron 10 of ROS1 at chromosome 6:117,646,698, which retains the ROS1 intracellular tyrosine kinase domain. Each gray row represents the sequencing read from a DNA fragment. Bottom bar shows the DNA sequence annotation of ROS1 (left) and EZR (right). [file CTM2-10-e196-s001.pptx]

## Slide 1
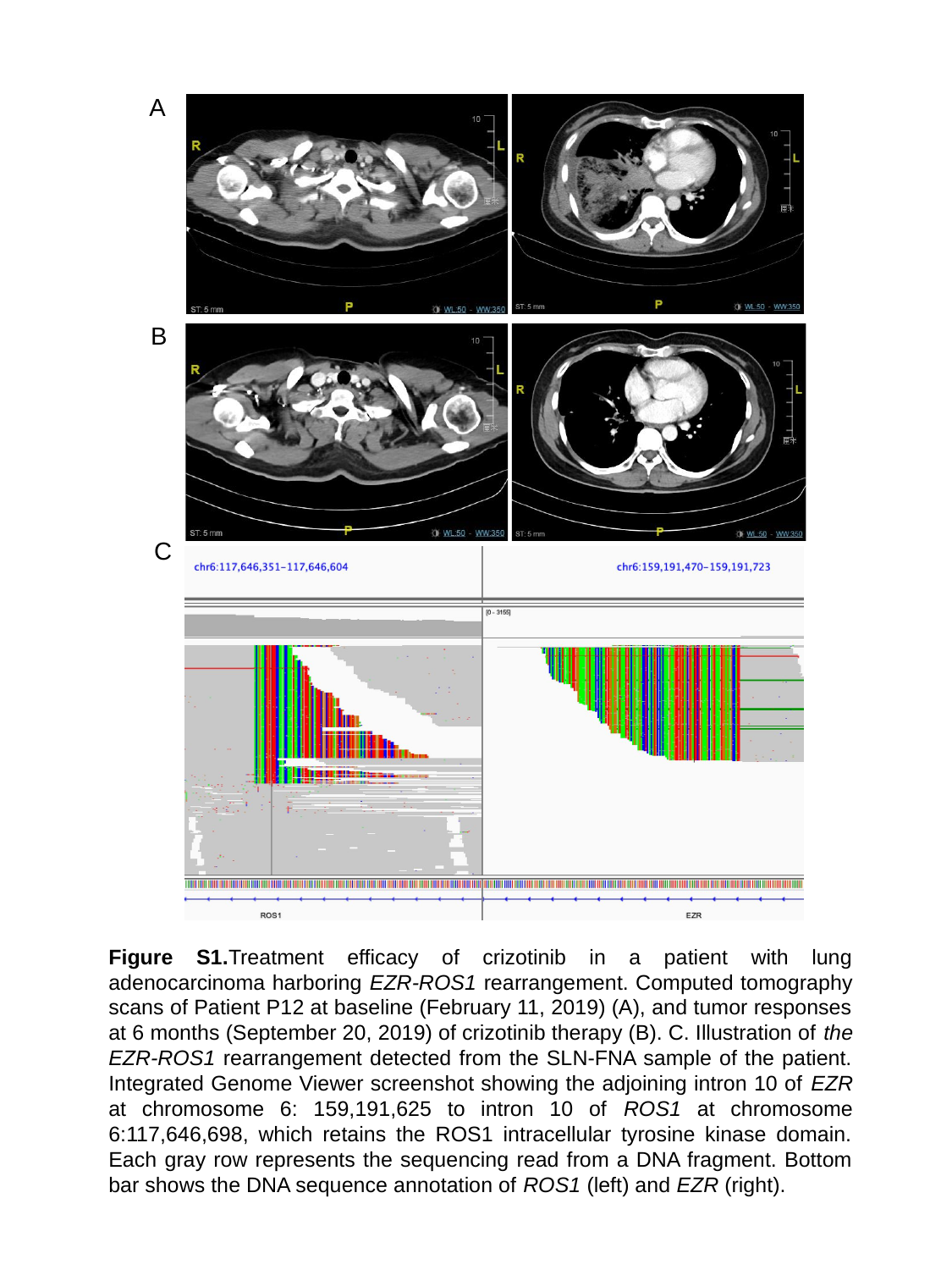

A
B
C
Figure S1.Treatment efficacy of crizotinib in a patient with lung adenocarcinoma harboring EZR-ROS1 rearrangement. Computed tomography scans of Patient P12 at baseline (February 11, 2019) (A), and tumor responses at 6 months (September 20, 2019) of crizotinib therapy (B). C. Illustration of the EZR-ROS1 rearrangement detected from the SLN-FNA sample of the patient. Integrated Genome Viewer screenshot showing the adjoining intron 10 of EZR at chromosome 6: 159,191,625 to intron 10 of ROS1 at chromosome 6:117,646,698, which retains the ROS1 intracellular tyrosine kinase domain. Each gray row represents the sequencing read from a DNA fragment. Bottom bar shows the DNA sequence annotation of ROS1 (left) and EZR (right).
